# Army Meteorological Register, for Twelve Years, from 1843 to 1854, Inclusive, Compiled from Observations Made by the Officers of the Medical Department of the Army, at the Military Posts of the United States

**Published:** 1856-03

**Authors:** 


ART. V.—*Army Meteorological Register, for twelve years, from* 1843 *to* 1854, *inclusive, compiled from observations made by the officers of the Medical Department of the Army, at the Military Posts of the United States.* Prepared under the direction of Brevet Brigadier General THOMAS LAWSON, Surgeon General U. S. A. Published by authority of Hon. JEFFERSON DAVIS, Secretary of War. Washington: 1855.

When the Medical Department of the Army was organized on its present basis, in 1821, Joseph Lovell was Surgeon General. A few years previous Secretary Calhoun had issued a system of regulations for observation of temperature and winds, at the suggestion of the Surgeon General. The earliest records on file in the office of the Surgeon General, date from the year 1819, and since that period, under the fostering care of General Lawson, the system has been extended, until every military post of the United States became an observatory of meteorological phenomena. Placed under the care of medical officers of great intelligence, with the reputation of the department depending on their fidelity, these observations are characterized by all the internal evidences of accuracy, and by breadth of detail.

The observations of the period previous to 1843, were published at its close, and we have now a volume giving the results of the remaining twelve years. In this volume we have only the observations of the thermometer, force and course of winds, clearness of sky, and inches of rain. Very properly, the results obtained from the barometer and hygrometer are reserved for another volume. As it is, we have a handsome quarto of 760 pages, printed in admirable style, and neatly bound.

The computations for such a work have been laborious. The supervision of the work was confided to Surgeon Richard H. Coolidge, assisted throughout by Lorin Blodget, Esqr. To this latter gentleman we are, says Surgeon Coolidge, indebted for "the general arrangement of the tables and the grouping of the stations in climatological districts. * * * The isothermal and rain charts were designed and prepared exclusively by him, and he is entitled to whatever of scientific value attaches to them, and to the accompanying report explanatory of the principles upon which they were constructed, and of the results which they exhibit."

We can expect to give only a faint idea of the labor involved in this splendid book. The whole number of posts at which observations have been made, for a greater or less period of time, is 158. The series includes observations of temperature, winds, and clouds, four times daily, viz: at sunrise, 9, A. M., 3, P. M., and 9, P. M.; and a daily observation of rain. The mean of the first three conditions, and the sum of the latter, is given for each month in the period observed. Such a series of observations, faithfully carried out over so vast an extent of territory, dotted by posts on two oceans, and in the heart of a great continent, and ranging from the tropics to the distant north, cannot fail to produce results valuable alike to the interests of medicine, or physics. Very much of the value of the reports will, however, depend upon their arrangement, and in this, we are happy to say, Prof. Blodget has been eminently successful. Only monthly means are given, these are again reduced to yearly means, and the final result is spread out to the eye at a glance in the elaborate yet distinct isothermal and hyetal charts.

On referring to the rain charts, we find a most satisfactory illustration of an assertion made in our report on "Hygrometric Conditions," to the American Medical Association, that "the map which would show the boundaries of those regions naturally warm and humid, would also circumscribe the fever districts, the favorite haunts of zymotic disease in this country, while every considerable elevation would mark a spot, dry in its atmosphere and healthy in its climate."

Turning to the chart we find a dark space extending from the mouth of the Red River to the Gulf, and thence easterly along the coast to a point beyond Mobile, where the annual precipitation of rain is 60 inches, and the mean annual temperature 70°. Here, then, in this low lying district, with five surface feet of water falling every year, and a yearly temperature equal to that of the summer at West Point or Chicago, we have a region whose ratio of mortality will range as high as its heats and its rains.

Going farther west on the same isothermal, we again recognize a yearly temperature of 70° to the west of the Rio del Norte, and south of El Paso. But here, though the temperature is equal to that of New Orleans, the amount of rain falling is but 20 inches, and a more healthy country does not exist. Lying beyond the western margin of the return trade-wind, it yields the same result of dryness to the hygrometer, as to the rain guage.

Within the field of the return trade-wind, however, we do not always find this same correspondence of a rainy sickly season, or a dry healthy one. The contrast afforded by the summers of 1854 and 1855, in the northern states, was remarkable. In 1854 a great drought prevailed, only six inches of rain falling during the three summer months. This drought was attended by wide-spread epidemics, and extraordinary fatality. In 1855, a different result obtained. During the three summer months nearly twelve inches of rain fell, and this succeeding a very wet spring. In spite of this double quantity of rain, the summer of 1855 was remarkably healthy. But this does not militate with the humid theory. The summer of 1854 gave a mean dew-point higher by many degrees than that of 1855. The water was not held in solution in the air, during the latter season, to exert its influence constantly upon the system, but was almost daily precipitated in rains, which each subtracted from the hygrometric condition.

The demands upon our time do not permit us at present to devote the labor necessary to develop the important results of this report. We propose, however, to give that labor at the earliest practicable time, and in the meantime we tender our thanks to Mr. Blodget for the beautiful volume under notice, and our congratulations on the high degree of success which has attended his labors upon it.

